# Effect of Changes in Photoperiods on Melatonin Expression and Gut Health Parameters in Laying Ducks

**DOI:** 10.3389/fmicb.2022.819427

**Published:** 2022-03-10

**Authors:** Yao-ming Cui, Jing Wang, Hai-jun Zhang, Guang-hai Qi, Han-zhen Qiao, Li-ping Gan, Shu-geng Wu

**Affiliations:** ^1^Laboratory of Quality and Safety Risk Assessment for Animal Products on Feed Hazards (Beijing) of the Ministry of Agriculture and Rural Affairs, Feed Research Institute, Chinese Academy of Agricultural Sciences, Beijing, China; ^2^College of Biological Engineering, Henan University of Technology, Zhengzhou, China

**Keywords:** intestinal morphology, melatonin, microbiota, laying duck, photoperiod

## Abstract

We investigated the effect of photoperiod on ileal morphology, barrier function, short-chain fatty acid (SCFA) contents, microbial flora, melatonin expression, and synthesis in laying ducks. After adaption, a total of 180 Jinding laying ducks (252 days old) were randomly divided into three treatments, receiving 12L (hours of light):12D (hours of darkness), 16L:8D, or 20L:4D. Each treatment had six replicates with 10 birds each. The formal experiment lasted 58 days. Compared with 12L:12D, the significantly higher values of villus height and goblet cell percentage (GCP) were observed in 16L:8D treatment, accompanied with the higher mRNA relative expression of zonula occludens-1, zonula occludens-2, zonula occludens-3, claudin-1, occludin, and mucin 2 (*P* < 0.05). Besides, significantly higher values of acetate and propionate, butyrate and total SCFA concentrations were simultaneously observed in ileal chyme of 16L:8D treatment (*P* < 0.05). For the ileal microbial community, the results of principal coordinate analysis (PCoA) visually presented that three photoperiod groups were mainly scattered into three clusters, indicating that the microbiota composition in different photoperiod treatments were quite dissimilar. Lower values of Shannon indicators were observed in the 20L:4D treatment (*P* < 0.05), meaning that the microbiota α-diversity decreased in the 20-h photoperiod. The relative abundance of Actinobacteria, Fusobacteria, and Proteobacteria at phylum level and *Fusobacterium*, *Clostridium_sensu_stricto_1*, and *Pectobacterium* at genus level kept an appropriate balance in the 16L:8D photoperiod. Melatonin level in serum decreased with the increasing photoperiods at 6:00 and 12:00, which was consistent with melatonin receptor expressions in the hypothalamus and ileal tissue. Meanwhile, the adenosine 3′,5′-cyclic phosphate (cAMP) contents were significantly downregulated in the pineal gland (*P* < 0.05), in response to the increase in photoperiod. In conclusion, an appropriate photoperiod could improve ileal morphology, barrier function, SCFA profile, and microbial flora, which may be attributed to the appropriate regulation of the circadian rhythm through melatonin as well as its receptor expression, and 16 h could be an adequate photoperiod for laying ducks.

## Introduction

Laying duck production is an important industry in China, as evidenced by a yield of 3,070,000 tons of eggs in 2018, equivalent to 42.3 billion yuan (Liu and Xu, 2019). The health conditions of ducks have attracted more and more attention in recent years. Among them, intestinal health has become a hot topic, which includes intestinal morphology, barrier function, short-chain fatty acid (SCFA) contents, etc. (Kogut, 2019). As is known to all, photoperiod is the most important environmental factor regulating the circadian rhythm of birds. During this process, melatonin serves as the most important regulator (Berra and Rizzo, 2009). Melatonin receptors have been observed in the ileum and colon of rodents (Chen et al., 2011), which indicated that intestinal motility and ecology could be affected by the circadian rhythm. However, most research paid attention to the effect of photoperiod on reproductive and productive performance (Chen et al., 2007; Geng et al., 2014), and there were almost no reports concerning the effect of photoperiod on intestinal health and microbial community. Therefore, more work is needed to evaluate the effect of photoperiod on intestinal morphology, barrier function, SCFA content, and microbial flora and to further explore the optimal photoperiod for intestinal health of laying ducks.

Photoperiod is supposed to be the most important environmental factor regulating the circadian rhythm of birds (Appenroth et al., 2021). Meanwhile, melatonin was regarded as the foremost modulator in the regulation of biological rhythm in birds (Berra and Rizzo, 2009). It can be speculated that photoperiod could probably have a huge effect on melatonin expression in birds. However, the current reports rarely involved the effects of different photoperiods on melatonin synthesis and secretion. Besides, massive changes in melatonin concentration over 1 day were observed in the plasma of quails (Voigt et al., 2016). To accurately evaluate the effect of photoperiod on the biological rhythm and melatonin expression in birds, the melatonin levels at different timepoints need to be investigated.

Melatonin (*N*-acetyl-5-methoxytryptamine) is synthesized and secreted in the pineal gland at night. Melatonin receptors have been observed in the hypothalamus of birds (Chowdhury et al., 2010), which contains the suprachiasmatic nuclei. The latter serves as a biological clock regulating the circadian rhythm. The physiological function exerted by melatonin on circadian rhythm regulation is mediated by its receptors (Wu et al., 2006). Hence, melatonin receptors should be investigated to further reveal the effect of photoperiod on the way that melatonin works. Moreover, the variation of melatonin expression in birds can be traced upstream to the change of the synthetic process in the pineal gland (Jiang et al., 2020). Melatonin synthesis mainly derives from tryptophan, forming 5-hydroxytryptophan, serotonin, and *N*-acetylserotonin and finally generating melatonin (Ge et al., 2021). This process is complicated, and the effect of changes in photoperiods on melatonin synthesis is still unclear. Therefore, more work is required to explore how different photoperiods impact the generation of melatonin in the pineal gland of laying birds.

The target of this research was to evaluate the effect of photoperiods from 12 to 20 h on intestinal morphology, barrier function, SCFA profile, and microbial flora in laying ducks. An appropriate photoperiod for Jinding laying ducks is expected to be obtained. Moreover, the expression of melatonin also needs to be investigated to explore the manner in which photoperiod regulates intestinal health through the biological rhythm.

## Materials and Methods

### Ethics Statement

All experimental protocols were approved by the Animal Care and Use Committee of the Feed Research Institute of the Chinese Academy of Agricultural Sciences (ACE-CAAS-20180915), and the methods were carried out in accordance with the relevant guidelines and regulations.

### Birds, Treatments, and Husbandry

A total of 180 Jinding laying ducks (252 days old) were randomly allocated into three treatments with a corn–soybean meal diet for 58 days (Cui et al., 2021a). Each group had six replicates with 10 ducks per replicate. An individual room (200 × 90 × 60 cm; length × width × height), containing adjustable light intensity, temperature, and ventilation as well as automatically controlled light timers (Cui et al., 2019), was prepared for each replicate. Birds received three lighting programs: 12L (hours of light):12D (hours of darkness), 16L:8D, and 20L:4D. During the light hours, all the ducks received light-emitting diode light with an average intensity of 20 (± 1.0) lux at eye level. A programmed ventilation of the whole aviary and cleaning of litters twice a day were used to guarantee air quality. Water and diet (in pellet form) were provided *ad libitum*, and feed intake was limited every day.

### Sample Collection

At 57 days of the treatment (309 days of age), the serum samples were collected at 0:00, 6:00, 12:00, and 18:00. At the end of the experiment (310 days of age), 12 ducks from each treatment were randomly selected (two birds per replicate) and quickly killed by an overdose of anesthesia (pentobarbital sodium). Segments (about 1.5 cm in length) in the middle portion of the ileum (approximately 5 cm from Meckel’s diverticulum) were collected, washed with PBS, and fixed in 10% neutral-buffered formalin for histology analysis. The remainder of the ileum was removed, opened longitudinally, and gently rinsed with PBS. The hypothalamus, pineal gland, ileal tissue, and chyme samples were collected, immersed in liquid nitrogen, and then stored at −80°C for subsequent measurement.

### Ileal Morphology

Ileal samples were washed, dehydrated, clarified, and embedded in paraffin. Serial sections were cut into 5 μm thickness, deparaffinized in xylene, rehydrated, stained with hematoxylin and eosin, fixed with neutral balsam, and observed by light microscopy (BX51, Olympus Co., Tokyo, Japan). The intestinal morphometry was evaluated by villus height (VH; from the tip of villus to the villus–crypt junction), crypt depth (CD; from the base up to the crypt–villus transition region), and the villus height-to-crypt depth ratio (VCR; Forte et al., 2016). The goblet cell percentage (GCP) was counted on 100 columnar cells of villus mucosa at × 400 magnification.

### Quantification of Melatonin Receptors and Tight Junction Protein With Real-Time PCR

Total RNA was obtained from the hypothalamus and ileal tissue samples using the TRIzol reagent (TIANGEN Biotech Co., Ltd., Beijing, China). The yield and integrity of RNA were measured with a NanoDrop 2000 spectrophotometer (Thermo Fisher Scientific, Waltham, MA) and agarose-ethidium bromide electrophoresis. Expression quantification was tested with a two-step reaction process, containing reverse transcription (RT) and PCR using a FastQuant RT kit (KR106, TIANGEN, Beijing, China), according to the description of Cui et al. (2019). The relative mRNA expression levels were normalized to avian β-actin with the 2^–ΔΔCt^ method (Livak and Schmittgen, 2001). Primer sequences of zonula occludens-1, zonula occludens-2, zonula occludens-3, claudin-1, claudin-2, occludin, mucin 2, melatonin receptor 1A, melatonin receptor 1B, and melatonin receptor 1C are detailed in Table 1. The β-actin was used to standardize the results because it was found that β-actin mRNA expression did not change significantly (*P* > 0.05) in response to different photoperiods (12L:12D, 16L:8D, and 20L:4D) in this research.

**TABLE 1:** Primer sequence of target and reference genes.

Gene	Forward primer (5′–3′)	Reverse primer (3′–5′)	GenBank number	Length (bp)
Zonula occludens-1	ACGCTGGTGAAATCAAGGAAGAA	AGGGACATTCAACAGCGTGGC	XM_013104936.1	255^a^
Zonula occludens-2	ACAGTGAAAGAAGCTGGCGTAG	GCTGTATTCCCTGCTACGGTC	XM_013093747.1	131^a^
Zonula occludens-3	CAACATCCCTGACATGGAAGACAT	TGTGTTCGTGTTGGTTGCGG	XM_005019888.2	187^a^
Claudin-1	TCATGGTATGGCAACAGAGTGG	CGGGTGGGTGGATAGGAAGT	XM_013108556.1	179^a^
Claudin-2	CTCCTCCTTGTTCACCCTCATC	GAACTCGCTCTTGGGTTTGTG	XM_005009661.2	160^a^
Occludin	CAGGATGTGGCAGAGGAATACAA	CCTTGTCGTAGTCGCTCACCAT	XM_013109403.1	160^a^
Mucin 2	GGGCGCTCAATTCAACATAAGTA	TAAACTGATGGCTTCTTATGCGG	XM_005024513.2	150^a^
Melatonin receptor 1A	TAGTGGCTTCTTGATGGG	AACAGGTTGGGCACGATA	NW_004676748.1	186^b^
Melatonin receptor 1B	GTGTATAGCTGCTGGAAC	CACAACAGTGATAGGGAC	NW_004676748.1	198^b^
Melatonin receptor 1C	ATCGCAATCAACCGCTAC	CAAGGACCCAACGAAGAA	NW_004676748.1	144^b^
β-Actin	AGAAATTGTGCGTGACATCAA	GGACTCCATACCCAAGAAAGAT	EF667345.1	227^a^

*^a^Sequences based on Wen et al. (2018). ^b^Sequences based on Feng et al. (2018).*

### Short-Chain Fatty Acid Profile

SCFA concentrations were measured according to the method of Wielen et al. (2020). Frozen ileal digesta (about 0.5 g) was thawed at 4°C and diluted fourfold with double-distilled water. After thawing, samples were centrifuged (10 min at 12,000 rpm), and 12.5 ml of a xylitol solution (0.586 M in 1.5 M HCl; internal standard) was added. Then, samples were centrifuged again (10 min at 12,000 rpm) before analysis using HPLC. Samples (20 μl) were injected into the HPLC with a Spark Holland autosampler (Emmen, the Netherlands). The HPLC was equipped with a Waters 2,996 Photodiode Array Detector and an organic acid column HPX-87H ion exclusion column (300 mm × 7.8 mm, Bio-Rad Laboratories Inc., Hercules, CA, United States). The column was operated at 40°C, with 5 mM NH_2_SO_4_ at 0.6 ml/min as the eluent.

### Melatonin Concentration in Serum

At the end of 309 days of age, two ducks from each group were fasted for 12 h, and then ∼3 ml of blood was extracted from a wing vein using evacuated tubes with coagulant. Blood sample acquisition was limited to 1 h, at 0:00, 6:00, 12:00, and 18:00. Serum samples were collected according to the method reported by Cui et al. (2019) and then stored at −20°C for the subsequent analysis. After thawing at 4°C overnight, the levels of melatonin were measured using ELISA kits for ducks (Shanghai Meilian, Bioengineering Institute, Shanghai, China; Li et al., 2015), with horseradish peroxidase marking the second antibody and tetramethylbenzidine serving as a chromogenic reagent.

### DNA Extraction and PCR Amplification of 16S rRNA Gene Sequences

Microbial DNA was extracted from ileal content samples (about 0.3 g) taken from laying ducks using the E.Z.N.A. Soil DNA Kit (Omega Bio-tek, Norcross, GA, United States). The integrity and quality of DNA samples were evaluated with 1% agarose gel electrophoresis and a NanoDrop D-1000 spectrophotometer (Thermo Fisher Scientific, Waltham, MA, United States). Microbial 16S rDNA sequences spanning the hypervariable regions v3–v4 were amplified using forward primer 338F (5′-ACTCCTACGGGA GGCAGCA-3′) and reverse primer 806R (5′-GGACTACHVGGGTWTCTAAT-3′). The PCR conditions were as follows: 2 min of denaturation at 95°C; 25 cycles containing denaturation at 95°C for 30 s, annealing at 55°C for 30 s, and extension at 72°C for 30 s; and a final extension of 5 min at 72°C. Amplicons were extracted from 2% agarose gels and purified using the AxyPrep DNA Gel Extraction Kit (Axygen Biosciences, Union City, CA, United States) to remove superabundant primer dimers and dNTPs. Purified amplicons were qualified and sequenced using the MiSeq platform at Beijing Biomarker Biotechnology Co., Ltd. (Beijing, China). The raw reads were deposited into the NCBI Sequence Read Archive (SRA) database (accession number: PRJNA761001).

### Metabolomic Profiling

On 310 days of age, pineal gland samples were collected from laying ducks, immersed in liquid nitrogen, and stored at −80°C before metabolite analysis. Metabolomic analysis was conducted using an ultraperformance liquid chromatography (UPLC) system (Waters Corporation, Milford, MA, United States) with a Waters Atlantis T-3 column (100 mm × 2.1 mm; 1.8 μm particle size) at 35°C and an injection volume of 5 μl. The UPLC system was equipped with a high-resolution tandem mass spectrometer Xevo G2-XS QTof (MS) (Waters Corporation, Milford, MA, United States). The mobile phases (flow rate of 0.5 ml/min) contained 0.1% formic acid (v/v) in double-distilled water (eluent A) and 0.1% formic acid (v/v) in acetonitrile (eluent B). The MS was adopted to test metabolites eluted from the column in both positive and negative ion modes. In the positive ion mode, the capillary and sampling cone voltages were 3.0 kV and 40.0 V, respectively. In the negative ion mode, the capillary and sampling cone voltages were 2.0 kV and 40.0 V, respectively. The mass spectrometry data were acquired through the centroid MSE mode. The scan time was 0.2 s, and the TOF mass range varied from 50 to 1,200 Da. Before MS/MS measurement, all precursors were made into fragments using 20–40 eV, and the scan time was 0.2 s. During the acquisition process, the LE signals were captured every 3 s to ensure mass accuracy. Besides, in order to assess the stability of the UPLC-MS during this acquisition process, a quality control sample was detected after every 10 samples.

### Statistical and Bioinformatic Analysis

Data of short-chain acids, morphology, and barrier melatonin in the ileum were analyzed with SAS, version 9.2. The homogeneity of variances and normality of the data were evaluated first. The Shapiro–Wilk test was adopted to measure the normality. Then, a one-way ANOVA and Duncan’s multiple range test were used for data analysis. Differences were considered to be statistically significant at *P* < 0.05. Data were presented as the mean and pooled SEM.

In microbial community analysis, raw paired-end sequences were conducted in Illumina HiSeq 2500. After sequencing, raw data were converted to raw reads through base calling. After filtration (Trimmomatic v0.33) and screening (Cutadapt 1.9.1), high-quality reads were obtained. Then, they were pieced through overlap (FLASH v1.2.7) to get clean reads. The chimera sequences were identified and removed to obtain effective reads by using the UCHIME (v4.2). The effective reads were clustered into operational taxonomic units (OTUs) with 97% sequence identity by Usearch (Edgar, 2013). Rarefaction curves and α-diversity analysis, containing Shannon, Simpson, ACE, and Chao1 indices, were calculated using QIIME 1. β-Diversity was estimated by computing the weighted UniFrac distance and visualized using principal coordinate analysis (PCoA), and the results were plotted using R package in R software (Liu et al., 2021). The significance of differentiation of microbial structure among treatments was assessed using the R package “microeco” (Liu et al., 2021).

In metabolite analysis, the raw data were corrected and filtrated by Progenesis QI software package (Progenesis QI v.2.2, Non-linear Dynamics, Newcastle, United Kingdom). Spectral deconvolution and normalization of the total ion amount generated a data matrix containing tR, m/z, and normalized peak area. The analytical variation was adjusted with the quality-control-based (QC) robust LOESS signal correction algorithm. All data were generalized logarithm-transformed and Pareto-scaled before multivariate statistical analysis, which contained an unsupervised principal component analysis (PCA) and orthogonal partial least squares discriminant analysis (OPLS-DA; R package ropls). The discrepant metabolites were determined through a variable importance in the projection (VIP) value > 1 of the OPLS-DA model and the *P*-values (< 0.05) from the Kruskal–Wallis test on the normalized peak intensities. Fold changes were calculated as the binary logarithm of average normalized peak intensity ratio between two treatments. HMDB and KEGG databases were used to confirm and annotate the differential metabolites.

## Results

### Ileal Morphology and Barrier Function

The changes in intestinal morphology and barrier indicators are listed in Tables 2, 3. Tight junction proteins, including zonula occludens-1 (*ZO-1*), zonula occludens-2 (*ZO-2*), zonula occludens-3 (*ZO-3*), claudin-1, claudin-2, and occludin, are intercellular–junctional molecules that could control intestinal permeability, thus restraining the entry of pathogens and maintaining intestinal health (Barekatain et al., 2019). Hence, the mRNA relative expressions of these tight junctions were investigated in this study. Different photoperiods had no significant effects on crypt depth, villus height/crypt depth, and the mRNA relative expression of claudin-2 (*P* > 0.05). Compared with the 12L:12D treatment, the significantly higher values of villus height and GCP were observed in 16L:8D, accompanied by the higher mRNA relative expression of *ZO-1*, *ZO-2*, claudin-1, occludin, and mucin 2 (*P* < 0.05). Besides, the significantly higher values of mRNA relative expression in *ZO-3* occurred in 16L:8D and 20L:4D treatments (*P* < 0.05), compared with the control.

**TABLE 2:** Effect of photoperiod on ileal morphology of laying ducks at 310 days of age.^1^

	Photoperiod^2^				
Items	12L:12D	16L:8D	20L:4D	SEM	*P*-value
Villus height (μm)	689.0^b^	788.6^a^	752.0^ab^	17.17	0.045
Crypt depth (μm)	197.7	199.4	203.0	3.65	0.85
Villus height/crypt depth	3.50	4.04	3.74	0.15	0.36
Goblet cell percentage (%)	16.80^b^	22.7^a^	20.8^ab^	1.04	0.049

*^1^Data are the mean of six replicates (two ducks in each replicate). ^2^L: hours of light; D: hours of darkness. ^a,b^Values within a row with no common superscripts differ significantly (P < 0.05).*

**TABLE 3:** Effect of photoperiod on the relative mRNA expression of tight junction protein and mucin 2 in the ileum of laying ducks (310 days of age).^1^

	Photoperiod^2^				
Items	12L:12D	16L:8D	20L:4D	SEM	*P*-value
Zonula occludens-1	1.00^b^	1.64^a^	1.33^ab^	0.095	0.014
Zonula occludens-2	1.00^b^	1.45^a^	1.19^ab^	0.076	0.041
Zonula occludens-3	1.00^b^	1.42^a^	1.53^a^	0.085	0.016
Claudin-1	1.00^b^	1.97^a^	1.41^ab^	0.143	0.011
Claudin-2	1.00	1.18	1.10	0.046	0.28
Occludin	1.00^b^	1.70^a^	1.29^ab^	0. 110	0.021
Mucin 2	1.00^b^	1.55^a^	1.23^ab^	0.083	0.013

*^1^Data are the mean of six replicates (two ducks in each replicate). ^2^L, hours of light; D, hours of darkness. ^a,b^Values within a row with no common superscripts differ significantly (P < 0.05).*

### Short-Chain Fatty Acids

The effects of photoperiod on SCFA concentrations in the ileum are shown in Table 4. No significant changes were observed in isobutyrate, valerate, and isovalerate concentrations (*P* > 0.05), in response to different photoperiods. Compared with those in the control (12L:12D), significantly higher values of acetate, propionate, butyrate, and total SCFA concentrations were simultaneously observed in 16L:8D treatment (*P* < 0.05).

**TABLE 4:** Effect of photoperiod on SCFA concentrations (μmol/g wet digesta) in the ileum of laying ducks (310 days of age).^1^

	Photoperiod^2^				
Items	12L:12D	16L:8D	20L:4D	SEM	*P*-value
Acetate	1.12^b^	1.31^a^	1.16^b^	0.03	0.045
Propionate	1.02^b^	1.20^a^	1.04^b^	0.03	0.037
Butyrate	0.61^b^	0.70^a^	0.59^b^	0.02	0.040
Isobutyrate	0.13	0.17	0.14	0.01	0.058
Valerate	0.18	0.20	0.17	0.01	0.52
Isovalerate	0.16	0.18	0.18	0.01	0.51
Total SCFA^3^	3.23^b^	3.77^a^	3.29^b^	0.07	0.001

*^1^Data are the mean of six replicates (two ducks in each replicate). ^2^L, hours of light; D, hours of darkness. ^3^Total SCFAs = acetate + propionate + butyrate + isobutyrate + valerate + isovalerate. ^a,b^Values within a row with no common superscripts differ significantly (P < 0.05).*

### Intestinal Microbial Diversity and Community

After filtering, an average of 57,262 reads per sample was obtained. The rarefaction curve for richness and numbers of shared OTUs were plotted to examine the sequencing depths. And most of the samples reached plateaus, which indicated that the sampling depth was adequate. The effect of photoperiod on ileal microbial α-diversity of laying ducks is detailed in Table 5. No significant differences in Simpson, ACE, and Chao1 indices were observed among all the treatments. Compared with those in 12L:12D, the significantly lower values of Shannon estimators were observed in the 20 h photoperiod (*P* < 0.05).

**TABLE 5:** Effect of photoperiod on microbial diversity in the ileum of laying ducks (310 days of age).^1^

	Photoperiod^2^				
Items	12L:12D	16L:8D	20L:4D	SEM	*P*-value
Shannon	4.31^a^	3.59^a^	2.27^b^	0.30	0.008
Simpson	0.85	0.78	0.68	0.03	0.13
ACE	302.3	293.9	307.7	9.71	0.82
Chao1	307.5	305.0	256.6	10.71	0.10

*^1^Data are the mean of six replicates (one duck in each replicate). ^2^L, hours of light; D, hours of darkness. ^a,b^Values within a row with no common superscripts differ significantly (P < 0.05).*

**FIGURE 1:**
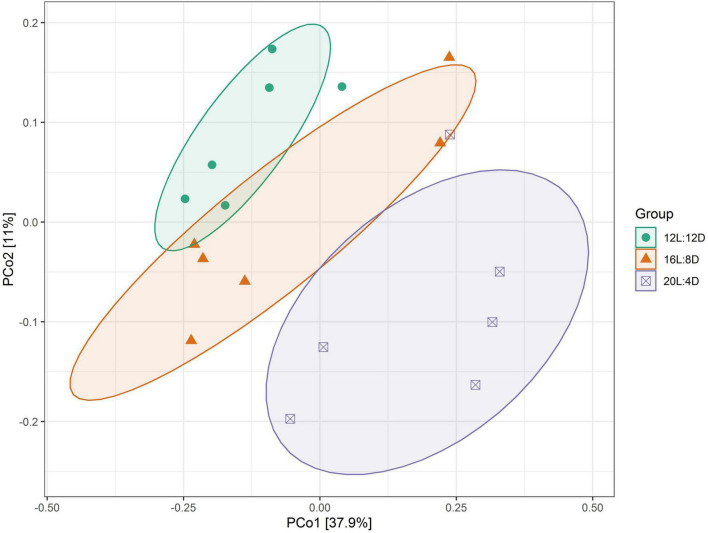
PCoA representing the similarity of ileal microbiota in laying ducks (310 days of age). Data are the mean of six replicates. L, hours of light; D, hours of darkness.

β-Diversity analysis was conducted to compare the microbial profiles among all the treatments as illustrated in Figure 1. PCoA was first performed to show a holistic perception of the microbiota. The results visually presented that these groups were mainly scattered into three clusters, which indicated that the microbiota compositions were quite dissimilar to each other.

**FIGURE 2:**
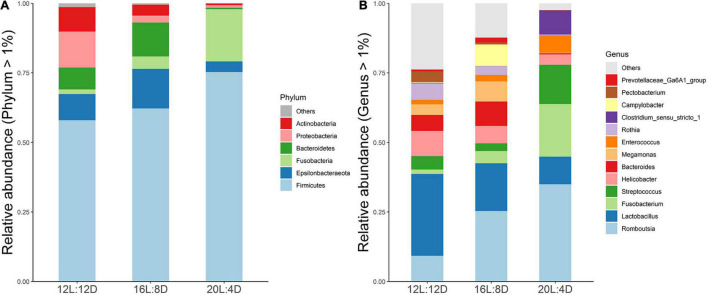
Composition of ileal microbiota of laying ducks (310 days of age) at genus **(A)** and phylum **(B)** levels. Data are the mean of six replicates. L, hours of light; D, hours of darkness.

To explore the effect of photoperiod on the bacterial community members of ileal microbiota, the taxonomic compositions were investigated at the phylum and genus levels. Six (Firmicutes, Epsilonbacteraeota, Fusobacteria, Bacteroidetes, Proteobacteria, and Actinobacteria) major phyla (relative abundance > 1%) dominated the bacterial community (Figure 2A). Meanwhile, these phyla could be allocated into 13 major genera (relative abundance > 1%; Figure 2B). Compared with the control (12L:12D), the significantly higher values of the Fusobacteria phylum and *Fusobacterium* and *Clostridium_sensu_stricto_1* genera occurred in 20L:4D (*P* < 0.05; Table 6). Besides, the significantly lower values of the Proteobacteria phylum and *Pectobacterium* genus were observed in both 16L:8D and 20L:4D treatments (*P* < 0.05), while the significantly lower relative abundance of the Actinobacteria phylum was observed in 20L:4D treatment (*P* = 0.020), compared with 12L:12D. There were no significant differences in the other microbes among these three treatments (*P* > 0.05).

**TABLE 6:** Effect of photoperiod on microbial relative abundance components in the ileum of laying ducks (310 days of age).^1^

	Photoperiod^2^				
Items	12L:12D	16L:8D	20L:4D	SEM	*P*-value
**Phylum (%)**					
Firmicutes	58.00	62.39	75.20	4.35	0.26
Epsilonbacteraeota	9.36	14.03	3.89	2.40	0.23
Fusobacteria	1.65^b^	4.41^b^	18.81^a^	2.44	0.002
Bacteroidetes	7.96	12.28	0.53	2.52	0.16
Proteobacteria	12.96^a^	2.54^b^	0.91^b^	2.01	0.018
Actinobacteria	8.64^a^	3.85^ab^	0.62^b^	1.25	0.020
**Genus**					
*Romboutsia*	9.21	25.37	34.86	5.52	0.16
*Lactobacillus*	29.54	17.20	9.88	4.45	0.20
*Fusobacterium*	1.62^b^	4.41^b^	18.81^a^	2.44	0.002
*Streptococcus*	4.72	2.76	14.12	3.02	0.28
*Helicobacter*	9.05	6.22	3.88	1.68	0.48
*Bacteroides*	5.80	8.67	0.39	1.81	0.17
*Megamonas*	3.76	7.28	0.21	1.79	0.29
*Enterococcus*	1.64	2.34	6.29	1.50	0.42
*Rothia*	5.85	3.07	0.24	0.98	0.056
*Clostridium_sensu_stricto_1*	0.15^b^	0.05^b^	8.66^a^	1.62	0.033
*Campylobacter*	0.31	7.81	0.00	1.71	0.10
*Pectobacterium*	3.83^a^	0.78^b^	0.14^b^	0.65	0.033
*Prevotellaceae_Ga6A1_group*	0.67	1.66	0.04	0.37	0.21

*^1^Data are the mean of six replicates (one duck in each replicate). ^2^L, hours of light; D, hours of darkness. ^a,b^Values within a row with no common superscripts differ significantly (P < 0.05).*

### Correlations Between Microbiota and Intestinal Morphology

**FIGURE 3:**
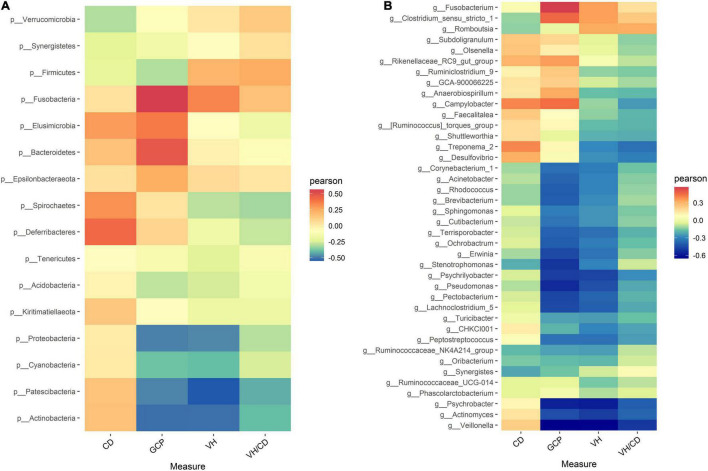
Heatmap of Spearman’s correlation between intestinal microbiota and intestinal morphology **(A)** at genus level, **(B)** at phylum level) in laying ducks (310 days of age). VH, villus height; CD, crypt depth; VH/CD, villus height/crypt depth; GCP, goblet cell percentage. Data are the mean of six replicates. L, hours of light; D, hours of darkness.

To investigate the intestinal bacteria associated with intestinal health, the correlations between the relative abundance of microbiota and intestinal morphology indicators were explored based on Spearman’s correlation coefficients (Figures 3A,B). The heatmap reflected that no significant correlations were observed between ileal morphology indicators (VH, CD, VH/CD, and GCP) and ileal bacteria at both phylum and genus levels (*P* > 0.05).

### Melatonin Concentration and Its Receptors mRNA Relative Expression

**FIGURE 4:**
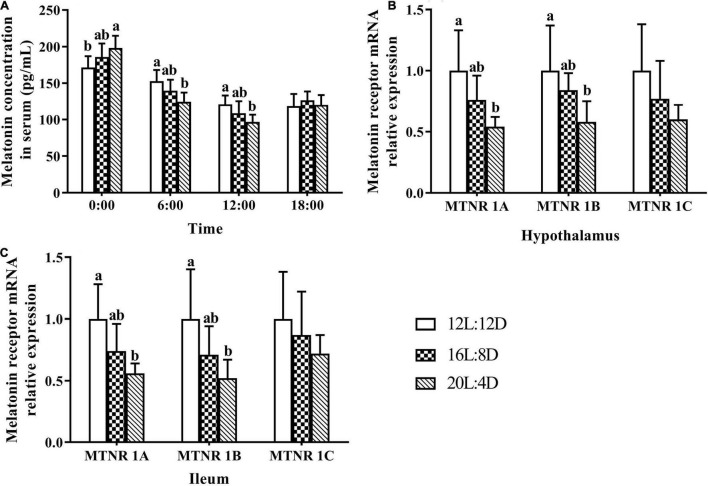
Melatonin content in serum **(A)** and its receptor relative mRNA expression in the hypothalamus **(B)** and ileum **(C)** in laying ducks (310 days of age). Data are the mean of six replicates (two ducks in each replicate). L, hours of light; D, hours of darkness. Data were expressed as mean ± SD. ^a,b^Values at the same timepoint or melatonin receptor type with no common superscripts differ significantly (*P* < 0.05).

Serum samples were extracted from laying ducks at 0:00, 6:00, 12:00, and 18:00 (309 days of age), and the melatonin concentrations in serum are demonstrated in Figure 4A. The melatonin concentrations changed significantly (*P* < 0.05) in response to the increasing photoperiods at 0:00, 6:00, and 12:00. The values of melatonin concentration numerically increased with increasing photoperiods at 0:00 and decreased at 6:00 and 12:00. Besides, compared with those in the control, the significantly higher levels of melatonin were observed in the 20L:4D group at 0:00 and 12:00 (*P* < 0.05); the significantly lower values of melatonin occurred in the 20L:4D group at 6:00 (*P* < 0.05).

The mRNA relative expression levels of melatonin receptors, including melatonin receptors (MTNR) 1A, 1B, and 1C, in response to different photoperiods can be seen in Figures 4B,C. Compared with that in the 12L:12D treatment, the MTNR (1A, 1B, and 1C) mRNA relative expression levels numerically increased with increasing photoperiods in the hypothalamus and ileal tissues. Furthermore, the significantly higher values of MTNR 1A and 1B were observed in 20L:4D treatments in both of these tissues (*P* < 0.05). No significant differences were observed in the mRNA relative expression levels of MTNR 1C (*P* > 0.05), in response to different photoperiods.

### Metabolomic Analysis

**FIGURE 5:**
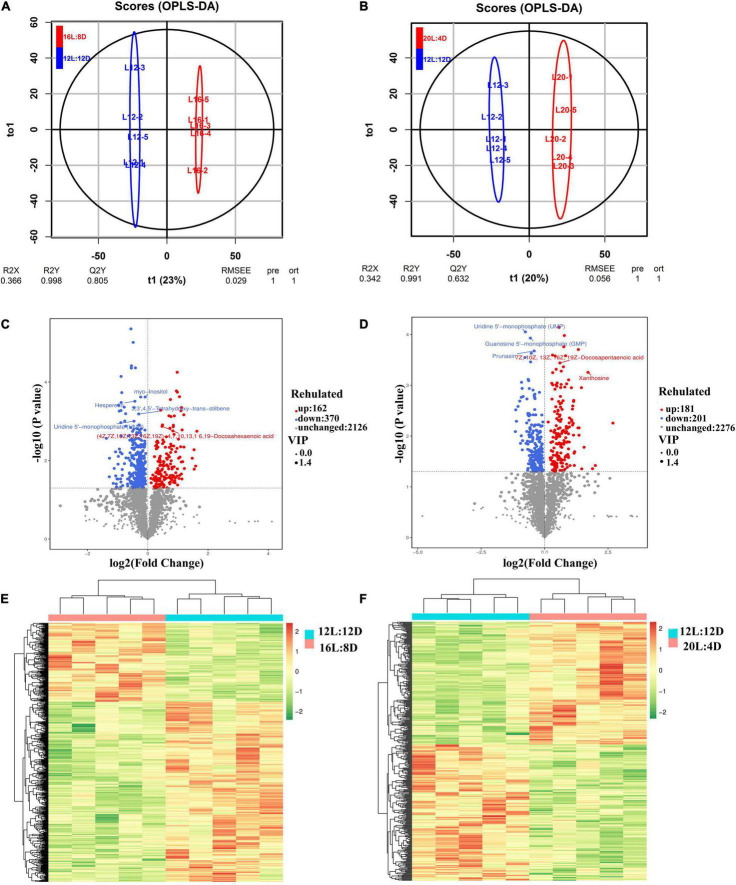
OPLS-DA **(A,B)**, volcano plot **(C,D)**, and clustered heatmap **(E,F)** of metabolites in the hypothalamus tissue of laying ducks (310 days of age). Data are the mean of five replicates. VIP, variable importance in projection; L, hours of light; D, hours of darkness.

To characterize the metabolite change responses to different photoperiods, LC-MS/MS-based metabolomic analysis in the pineal gland from laying ducks was conducted. A total of 2,658 metabolites were analyzed in this study. Based on OPLS-DA analysis, R2Y were 0.998 and 0.991, while Q2Y were 0.805 and 0.632 (Figures 5A,B), which indicated that this model was effective and reliable and VIP could be used in discrepant metabolite screening. The filter criteria were as follows: *P* < 0.05 and VIP > 1. Based on these conditions, 162 metabolites were significantly upregulated and 370 metabolites were significantly downregulated in 16L:8D, compared with 12L:12D (Figure 5C). Meanwhile, 181 metabolites were significantly upregulated and 201 metabolites were significantly downregulated in 16L:8D (Figure 5D). Furthermore, clustered heatmaps based on discrepant metabolites showed that samples in one treatment (including 12L:12D, 16L:8D, and 20L:4D) occurred in the same cluster through clustering. And the metabolites of samples from the same treatment exhibited good similarity (Figures 5E,F).

The differential metabolites among different photoperiods in the pineal gland of laying ducks are demonstrated in Table 7. Compared with that in the control (12L:12D), adenosine 3′,5′-cyclic phosphate and adenosine monophosphate were significantly downregulated in 16L:8D and 20L:4D (*P* < 0.05, VIP > 1). Furthermore, adenosine 3′,5′-cyclic phosphate is a crucial factor in the synthesis pathway of melatonin.

**TABLE 7:** Differential metabolites in the pineal gland of laying ducks (310 days of age) among different photoperiods.^a^

Differential metabolites	Fold change	*P*-value	VIP^b^	Regulated
**12L:12D VS 16L:8D^c^**				
Adenosine	1.467	0.026	1.487	Up
Adenosine 3′,5′-cyclic phosphate	0.784	0.023	1.534	Down
Adenosine monophosphate	0.797	0.013	1.605	Down
**12L:12D VS 20L:4D**				
Adenosine	0.903	0.016	1.629	Down
Adenosine 3′,5′-cyclic phosphate	0.700	0.006	1.809	Down
Adenosine monophosphate	0.671	0.007	1.869	Down

*^a^Data are the mean of five replicates (one duck in each replicate). ^b^VIP, variable importance in projection. ^c^L, hours of light; D, hours of darkness.*

## Discussion

In the year 2021, we reported the effect of photoperiod on laying performance, ovarian morphology, reproductive hormone secretion and its receptor gene mRNA expression, eggshell quality, and the quality characteristics of the tibia, femur, and ulna in ducks during the laying phase (Cui et al., 2021a,b). Independent from previous publications, this study reported the effect of photoperiod on ileal morphology, barrier function and microbiota, and melatonin expression and synthesis in laying ducks. The photoperiod serves as a crucial environmental factor in poultry production and health, which has biological and physiological significance through regulating the circadian rhythm and changing the time for rest or regeneration (Malleau et al., 2007). However, most of the current research concerning photoperiod in birds focused on health and productivity (Chen et al., 2007). There are very few reports involving the effect of photoperiod on intestinal morphology, barrier function, and microbiota. Meanwhile, melatonin receptor expression levels were observed in intestinal tissue (Feng et al., 2018), which indicated that intestinal morphology and microbiota could probably be affected by different photoperiods, due to melatonin serving as the most important hormone in circadian rhythm regulation. Hence, changes of intestinal morphology in response to different photoperiods were investigated, and the superior intestinal morphology indicators occurred in 16L:8D treatment, evidenced by the higher values of villus height and GCP. Intestinal morphology is supposed to be closely related to animal performance because an expanded absorptive surface area means greater digestion and absorption, which was consistent with the results in our previous report that higher average daily feed intake was observed in 16L:8D treatment compared with the control (Cui et al., 2021a). Goblet cells are differentiated from intestinal epithelial stem cells and play a vital role in intestinal mucosal barrier maintenance through the secretion of mucus (Knoop and Newberry, 2018). Among mucus, mucin 2 is the main component, which plays a crucial role in the colonization process of intestinal beneficial bacteria (Johansson et al., 2011). Hence, a higher GCP meant superior intestinal health status. Besides, the ileal barrier function in response to different photoperiods was also investigated in this study, and the tight junction proteins (including ZO-1, ZO-2, ZO-3, claudin-1, and occludin) and mucin 2 were upregulated in 16L:8D treatment. Tight junction proteins are intercellular–junctional molecules that could control intestinal permeability, thus restraining the entry of pathogens and maintaining intestinal health (Barekatain et al., 2019). Hence, higher tight junction protein expression meant more superior morphology and health status of the intestine. A higher level of mucin 2 expression was consistent with more goblet cell numbers in ileal tissue, which indicated that more beneficial microorganisms could colonize in the ileal tract. Based on the ileal morphology and barrier indicators, 16L:8D was supposed to be an appropriate photoperiod for laying ducks.

Short-chain acids serve as the main fuel for the proliferation and differentiation of intestinal epithelial stem cells (Park et al., 2016), which is supposed to be beneficial for the intestinal morphology and barrier (Xie et al., 2020). Thus, the changes of SCFA concentrations in response to different photoperiods were further investigated in this research. Higher levels of acetate, propionate, butyrate, and total SCFAs in ileal digesta were observed in 16L:8D treatment, which was consistent with the higher values of villus height and GCP occurring in this group. These results indicated that an increase in SCFAs might be responsible for the improvement in ileal morphology, which was consistent with the results of Liao et al. (2020). The improved SCFA content may be due to the optimization of microbiota in the intestine (LeBlanc et al., 2017), while the increased goblet cell number and mucin 2 expression could create the conditions for the colonization of beneficial intestinal bacteria.

Hence, the changes of intestinal microbiota in response to different photoperiods were investigated in this study. In the α-diversity analysis, the Shannon and Simpson estimators were adopted to evaluate the microbiota diversity, while the ACE and Chao1 indices reflected the microbiota richness (Ballou et al., 2016). In our results, lower values of Shannon indicators were observed in the 20L:4D treatment, which meant that the microbiota α-diversity in the ileum of laying ducks would decrease when the photoperiod reached 20 h/day. A higher level of species diversity means a more stable microbiota community, which can prevent the colonization of pathogens and thus will be beneficial to the productivity of the host bird (Han et al., 2017). Hence, lower microbiota α-diversity indicated poor productivity, which was consistent with a previous report that the productive performance of laying ducks significantly decreased in the 20L:4D treatment (Cui et al., 2021a). In addition, PCoA was adopted to investigate the β-diversity of ileal microbiota. The results showed significant clustering according to experimental groups, which demonstrated that the ileal microbial community structure was significantly affected by different photoperiods. Therefore, we speculated that the ileal microbiota of laying ducks could probably vary significantly in response to different photoperiods, and variations in microbial composition and some specific taxon in this present research were further analyzed. Data showed that, at the phylum level of ileal microbial composition, Fusobacteria increased, while Proteobacteria and Actinobacteria decreased, with the increasing photoperiods from 12 to 20 h. Given the adverse effects of Fusobacteria and Proteobacteria as well as the beneficial effect of Actinobacteria (Shin et al., 2015; Kelly et al., 2018; Massot-Cladera et al., 2020), these results indicated that an appropriate photoperiod between 12 and 20 h probably had a balanced ileal microbiota composition, including helpful and unhelpful microorganisms. Ileal microbial composition at the genus level exhibited a similar law, as evidenced by the relative increase in *Fusobacterium* and *Clostridium_sensu_stricto_1* (supposed to be unbeneficial) and decrease in Pectobacterium (supposed to be beneficial) with increasing photoperiods from 12 to 20 h (Ma et al., 2018; Wang et al., 2018; Hertel et al., 2021). Hence, a 16 h photoperiod could be considered an appropriate photoperiod between 12 and 20 h. Moreover, *Bacteroides* and *Megamonas* were the preponderant genera in the 16L:8D photoperiod treatment, and they exhibited a positive correlation with higher production of propionate and butyrate (Yang et al., 2013; Liang et al., 2021). These findings coincided with the above results that the 16L:8D photoperiod treatment had better SCFA production. Taking the ileal morphology, barrier, SCFA concentrations, and microbial composition into consideration, 16 h of light time per day could be a suitable photoperiod for laying ducks.

Change of intestinal microbial community may be due to the variation in the biological rhythm (Voigt et al., 2016). However, the level of melatonin in serum was reported to vary with time for 1 day (Chowdhury et al., 2010). Hence, melatonin levels in serum at four timepoints during 1 day were explored in this research, and the results showed that the melatonin contents changed significantly in response to different photoperiods. The physiological actions of melatonin are mainly mediated by its receptors (Wu et al., 2006). Hence, the expression levels of melatonin receptors in the hypothalamus and ileal tissue were also investigated in the present study. The consistent decrease of melatonin receptor expressions with the increasing photoperiods was observed in the hypothalamus and ileal tissue, which was similar to the level of melatonin variation in serum at 6:00 and 12:00 a.m. Given that the hypothalamus and ileal tissue were collected from birds between 10:00 and 11:00 a.m., these results may be due to the fact that hormone receptors could be induced by the release of the hormone itself (Ing and Tornesi, 1997). The level of melatonin increased with increasing photoperiods from 12 to 20 h at 0:00 and decreased at 6:00 and 12:00 in this research, and 16 h may be a moderate photoperiod for melatonin synthesis and secretion. Besides, melatonin was supposed to stimulate the release of the gonadotropin-inhibitory hormone by the avian hypothalamus (Chowdhury et al., 2010), which was coincident with our previous report (Cui et al., 2021a). In that study, the gonadotropin-inhibitory hormone increased from 12 to 20 h photoperiods (sample collection between 11:00 and 12:00), while melatonin levels decreased from 12 to 20 h photoperiods at 12:00 in this study. The changes of the gonadotropin-inhibitory hormone as well as other reproductive hormones in response to different photoperiods (Cui et al., 2021a) also indicated that the changes of melatonin in response to different photoperiods in the current study could be reliable. As is known to all, melatonin was supposed to be the most important hormone in the circadian rhythm regulation of birds (Berra and Rizzo, 2009). Hence, the appropriate level of melatonin and its receptors meant a suitable circadian rhythm for laying ducks. It can be speculated that the superior ileal morphology, barrier function, SCFA concentration, and microbial community in the 16L:8D photoperiod could be due to the appropriate circadian rhythm regulated by melatonin.

Melatonin was supposed to be mainly synthesized and secreted in the pineal gland of birds (Jiang et al., 2020). Hence, the regulation of melatonin generation by photoperiod was further investigated in the pineal gland using metabonomics in this research. The results showed that the concentrations of AMP and cAMP were significantly downregulated in the pineal gland of laying ducks with the increasing photoperiods from 12 to 20 h. These results indicated that the increase in light time could decrease the level of cAMP in the pineal gland of ducks. Reduction of cAMP could cause the downregulation of key enzyme synthesis (such as Aanat) in the pinealocyte and reduce the generation of *N*-acetylserotonin (Ge et al., 2021). The latter is a crucial precursor for melatonin synthesis. Therefore, the increase in melatonin level could be ascribed to the decrease in cAMP level with the increasing photoperiods.

## Conclusion

In conclusion, an increment in photoperiod could improve ileal morphology, barrier function, microbial flora, and SCFA profile, and 16L:8D was supposed to be the appropriate photoperiod for laying ducks. These results may be attributed to the moderate expression of melatonin and its receptors, and the increase in melatonin could further be traced upstream to the decreased level of cAMP in the pineal gland with the increasing photoperiods.

## Data Availability Statement

The datasets presented in this study can be found in online repositories. The names of the repository/repositories and accession number(s) can be found below: https://www.ncbi.nlm.nih.gov/, PRJNA761001.

## Ethics Statement

The animal study was reviewed and approved by the Animal Care and Use Committee of the Feed Research Institute of the Chinese Academy of Agricultural Sciences (ACE-CAAS-20180915). Written informed consent was obtained from the owners for the participation of their animals in this study.

## Author Contributions

S-gW and Y-mC conceived and designed the experiments. Y-mC performed animal experiments, analyzed the data, and wrote the manuscript. S-gW assisted with data analysis and manuscript writing. G-hQ, JW, H-jZ, H-zQ, and L-pG supervised and provided continuous guidance for the experiment. All authors discussed the results, reviewed the manuscript, read, and approved the final manuscript.

## Conflict of Interest

The authors declare that the research was conducted in the absence of any commercial or financial relationships that could be construed as a potential conflict of interest.

## Publisher’s Note

All claims expressed in this article are solely those of the authors and do not necessarily represent those of their affiliated organizations, or those of the publisher, the editors and the reviewers. Any product that may be evaluated in this article, or claim that may be made by its manufacturer, is not guaranteed or endorsed by the publisher.

## Abbreviations

SCFAs: short-chain fatty acids
L: hours of light
D: hours of darkness
cAMP: adenosine 3′,5′-cyclic phosphate
VH: villus height
CD: crypt depth
VCR: the villus height-to-crypt depth ratio
GCP: goblet cell percentage
*ZO1*: zonula occludens-1
*ZO2*: zonula occludens-2
*ZO3*: zonula occludens-3.

## References

[B1] Appenroth D. Wagner G. C. Hazlerigg D. G. West A. C. (2021). Evidence for circadian-based photoperiodic timekeeping in Svalbard ptarmigan, the northernmost resident bird. *Curr. Biol.* 31 2720–2727. 10.1016/j.cub.2021.04.009 33930302

[B2] Ballou A. L. Ali R. A. Mendoza M. A. Ellis J. Hassan H. M. Croom W. (2016). Development of the chick microbiome: how early exposure influences future microbial diversity. *Front. Vet. Sci.* 3:2. 10.3389/fvets.2016.00002 26835461 PMC4718982

[B3] Barekatain R. Chrystal P. V. Howarth G. S. Mclaughlan C. J. Gilani S. Nattrass G. S. (2019). Performance, intestinal permeability, and gene expression of selected tight junction proteins in broiler chickens fed reduced protein diets supplemented with arginine, glutamine, and glycine subjected to a leaky gut model. *Poult. Sci.* 98 6761–6771. 10.3382/ps/pez393 31328774 PMC6869755

[B4] Berra B. Rizzo A. M. (2009). Melatonin: circadian rhythm regulator, chronobiotic, antioxidant and beyond. *Clin. Dermatol.* 27 202–209. 10.1016/j.clindermatol.2008.04.003 19168001

[B5] Chen C. Q. Fichna J. Bashashati M. Li Y. Y. Storr M. (2011). Distribution, function and physiological role of melatonin in the lower gut. *World J. Gastroenterol.* 17 3888–3898. 10.3748/wjg.v17.i34.3888 22025877 PMC3198018

[B6] Chen H. Huang R. L. Zhang H. X. Di K. Q. Pan D. Hou Y. G. (2007). Effects of photoperiod on ovarian morphology and carcass traits at sexual maturity in pullets. *Poult. Sci.* 86 917–920. 10.1093/ps/86.5.917 17435026

[B7] Chowdhury V. S. Yamamoto K. Ubuka T. Bentley G. E. Hattori A. Tsutsui K. (2010). Melatonin stimulates the release of gonadotropin-inhibitory hormone by the avian hypothalamus. *Endocrinology* 151 271–280. 10.1210/en.2009-0908 19952272

[B8] Cui Y. M. Wang J. Zhang H. J. Feng J. Wu S. G. Qi G. H. (2019). Effect of photoperiod on ovarian morphology, reproductive hormone secretion and hormone receptor mRNA expression in layer ducks during the pullet phase. *Poult. Sci.* 98 2439–2447. 10.3382/ps/pey601 30668853

[B9] Cui Y. M. Wang J. Zhang H. J. Qi G. H. Wu S. G. (2021a). Effect of photoperiod on performance, ovarian morphology, reproductive hormone level, and hormone receptor mRNA expression in laying ducks. *Poult. Sci.* 100:100979. 10.1016/j.psj.2021.01.002 33677400 PMC8046941

[B10] Cui Y. M. Wang J. Zhang H. J. Qi G. H. Wu S. G. (2021b). Effect of photoperiod on eggshell quality and quality characteristics of tibia, femur and ulna in laying ducks. *Poult. Sci.* 100:101376. 10.1016/j.psj.2021.101376 34391963 PMC8371216

[B11] Edgar R. C. (2013). UPARSE: highly accurate OTU sequences from microbial amplicon reads. *Nat. Methods* 10 996–998. 10.1038/NMETH.2604 23955772

[B12] Feng P. S. Zhao W. Q. Xie Q. Zeng T. Lu L. Z. Yang L. (2018). Polymorphisms of melatonin receptor genes and their associations with egg production traits in Shaoxing duck. *Asian Australas J. Anim. Sci.* 31 1535–1541. 10.5713/ajas.17.0828 29642678 PMC6127595

[B13] Forte C. Acuti G. Manuali E. Proietti P. C. Pavone S. Trabalzamarinucci M. (2016). Effects of two different probiotics on microflora, morphology, and morphometry of gut in organic laying hens. *Poult. Sci.* 95 2528–2535. 10.3382/ps/pew164 27143778

[B14] Ge W. Yan Z. H. Wang L. Tan S. J. Liu J. Reiter R. J. (2021). A hypothetical role for autophagy during the day/night rhythm-regulated melatonin synthesis in the rat pineal gland. *J. Pineal Res.* 71:e12742. 10.1111/jpi.12742 33960014

[B15] Geng A. L. Xu S. F. Zhang Y. Zhang J. Chu Q. Liu H. G. (2014). Effects of photoperiod on broodiness, egg-laying and endocrine responses in native laying hens. *Br. Poult. Sci.* 55 264–269. 10.1080/00071668.2013.878782 24404878

[B16] Han Z. Willer T. Li L. Pielsticker C. Rychlik I. Velge P. (2017). Influence of the gut microbiota composition on Campylobacter jejuni colonization inchickens. *Infect. Immun.* 85 e380–e417. 10.1128/IAI.00380-17 28808158 PMC5649013

[B17] Hertel J. Heinken A. Martinelli F. Thiele I. (2021). Integration of constraint-based modeling with fecal metabolomics reveals large deleterious effects of *Fusobacterium* spp. on community butyrate production. *Gut Microbes* 13:e1915673. 10.1080/19490976.2021.1915673 34057024 PMC8168482

[B18] Ing N. H. Tornesi M. B. (1997). Estradiol up-regulates estrogen receptor and progesterone receptor gene expression in specific ovine uterine cells. *Biol. Reprod.* 56 1205–1215. 10.1095/biolreprod56.5.1205 9160720

[B19] Jiang N. Cao J. Wang Z. X. Dong Y. L. Chen Y. X. (2020). Effect of monochromatic light on the temporal expression of N-acetyltransferase in chick pineal gland. *Chronobiol. Int.* 37 1140–1150. 10.1080/07420528.2020.1754846 32308045

[B20] Johansson M. E. Larsson J. M. H. Hansson G. C. (2011). The two mucus layers of colon are organized by the MUC2 mucin, whereas the outer layer is a legislator of host-microbial interactions. *PNAS* 108 4659–4665. 10.1073/pnas.1006451107 20615996 PMC3063600

[B21] Kelly D. Yang L. Y. Pei Z. H. (2018). Gut microbiota, fusobacteria, and colorectal cancer. *Diseases* 6:109. 10.3390/diseases6040109 30544946 PMC6313651

[B22] Knoop K. A. Newberry R. D. (2018). Goblet cells: multifaceted players in immunity at mucosal surfaces. *Mucosal Immunol.* 11 1551–1557. 10.1038/s41385-018-0039-y 29867079 PMC8767637

[B23] Kogut M. K. (2019). The effect of microbiome modulation on the intestinal health of poultry. *Anim. Feed Sci. Tech.* 250 32–40. 10.1016/j.anifeedsci.2018.10.008

[B24] LeBlanc J. G. Chain F. Martín R. Bermúdez-Humarán L. G. Courau S. Langella P. (2017). Beneficial effects on host energy metabolism of short-chain fatty acids and vitamins produced by commensal and probiotic bacteria. *Microb. Cell Fact.* 16:79. 10.1186/s12934-017-0691-z 28482838 PMC5423028

[B25] Li J. Cao J. Wang Z. X. Dong Y. L. Chen Y. X. (2015). Melatonin plays a critical role in inducing B lymphocyte proliferation of the bursa of Fabricius in broilers via monochromatic lights. *J. Photoch. Photobio. B.* 142 29–34. 10.1016/j.jphotobiol.2014.11.002 25496874

[B26] Liang D. Li N. Dai X. F. Zhang H. Hu H. H. (2021). Effects of different types of potato resistant starches on intestinal microbiota and short-chain fatty acids under in vitro fermentation. *Int. J. Food Sci. Tech.* 56 2432–2442. 10.1111/ijfs.14873

[B27] Liao X. D. Shao Y. X. Sun G. M. Yang Y. F. Zhang L. Y. Guo Y. L. (2020). The relationship among gut microbiota, short-chain fatty acids, and intestinal morphology of growing and healthy broilers. *Poult. Sci.* 99 5883–5895. 10.1016/j.psj.2020.08.033 33142506 PMC7647869

[B28] Liu C. Cui Y. M. Li X. Z. Yao M. J. (2021). Microeco: an R package for data mining in microbial community ecology. *FEMS Microbiol. Ecol.* 97:fiaa255. 10.1093/femsec/fiaa255 33332530

[B29] Liu L. Z. Xu Q. (2019). Evolution trend. problems and development strategies of waterfowl industry market. *China Poultry.* 41 1–7. 10.16372/j.issn.1004-6364.2019.20.001

[B30] Livak K. J. Schmittgen T. D. (2001). Analysis of relative gene expression data using real-time quantitative PCR and the 2^Δ^ ^Δ^ ^CT^ Method. *Methods* 25 402–408. 10.1006/meth.2001.1262 11846609

[B31] Ma X. W. Hu Y. C. Li X. Zheng X. T. Wang Y. T. Zhang J. M. (2018). *Periplaneta americana* ameliorates dextran sulfate sodium-induced ulcerative colitis in rats by keap1_Nrf-2 activation, intestinal barrier function, and gut microbiota regulation. *Front. Pharmacol.* 9:00944. 10.3389/fphar.2018.00944 30186174 PMC6113651

[B32] Malleau A. E. Duncan I. J. H. Widowskia T. M. Atkinsona J. L. (2007). The importance of rest in young domestic fowl. *Appl. Anim. Behav. Sci.* 106 52–69. 10.1016/j.applanim.2006.06.017

[B33] Massot-Cladera M. Azagra-Boronat I. Franch À Castell M. Rodríguez-Lagunas M. J. Pérez-Cano F. J. (2020). Gut health-promoting benefits of a dietary supplement of vitamins with inulin and acacia fibers in rats. *Nutrients* 12:2196. 10.3390/nu12082196 32718017 PMC7468733

[B34] Park J. Kotani T. Konno T. Setiawan J. Kitamura Y. Imada S. (2016). Promotion of intestinal epithelial cell turnover by commensal bacteria: role of short-chain fatty acids. *PLoS One* 11:e0156334. 10.1371/journal.pone.0156334 27232601 PMC4883796

[B35] Shin N. R. Whon T. W. Bae J. W. (2015). *Proteobacteria*: microbial signature of dysbiosis in gut microbiota. *Trends Biotechnol*. 33 496–503. 10.1016/j.tibtech.2015.06.011 26210164

[B36] Voigt R. M. Summa K. C. Forsyth C. B. Green S. J. Engen P. Naqib A. (2016). The circadian clock mutation promotes intestinal dysbiosis. *Alcohol. Clin. Exp. Res.* 40 335–347. 10.1111/acer.12943 26842252 PMC4977829

[B37] Wang J. Ji H. F. Wang S. X. Liu H. Zhang W. Zhang D. Y. (2018). Probiotic *Lactobacillus plantarum* promotes intestinal barrier function by strengthening the epithelium and modulating gut microbiota. *Front. Microbiol.* 9:01953. 10.3389/fmicb.2018.01953 30197632 PMC6117384

[B38] Wen M. Zhao H. Liu G. M. Chen X. L. Wu B. Tian G. (2018). Effect of zinc supplementation on growth performance, intestinal development, and intestinal barrier-related gene expression in pekin ducks. *Biol. Trace. Elem. Res.* 183 351–360. 10.1007/s12011-017-1143-7 28895044

[B39] Wielen P. W. J. J. Biesterveld S. Notermans S. Hofstra H. Urlings B. A. P. Knapen F. V. (2020). Role of volatile fatty acids in development of the cecal microflora in broiler chickens during growth. *Appl. Environ. Microb.* 66 2536–2540. 10.1128/AEM.66.6.2536-2540.2000 10831435 PMC110578

[B40] Wu Y. H. Zhou J. N. Balsar R. Unmehopa U. Bao A. Jockers R. (2006). Distribution of MT1 melatonin receptor immunoreactivity in the human hypothalamus and pituitary gland: colocalization of MT1 with vasopressin, oxytocin, and corticotropin-releasing hormone. *J. Comp. Neurol.* 499 897–910. 10.1002/cne.21152 17072839

[B41] Xie Y. Ding F. Di W. J. Lv Y. F. Xia F. Sheng Y. L. (2020). Impact of a high−fat diet on intestinal stem cells and epithelial barrier function in middle−aged female mice. *Mol. Med. Rep.* 21 1133–1144. 10.3892/mmr.2020.10932 32016468 PMC7003032

[B42] Yang J. Y. Martínez I. Walter J. Keshavarzian A. Rose D. J. (2013). In vitro characterization of the impact of selected dietary fibers on fecal microbiota composition and short chain fatty acid production. *Anaerobe* 23 74–81. 10.1016/j.anaerobe.2013.06.012 23831725

